# Self-Care Activities Among Diabetic Patients in an Urban Health Centre in Thirumazhisai, Tamil Nadu: A Cross-Sectional Study

**DOI:** 10.7759/cureus.68634

**Published:** 2024-09-04

**Authors:** Priya P, Srivaishnavi KN

**Affiliations:** 1 Community Medicine, Saveetha Medical College and Hospital, Saveetha Institute of Medical and Technical Sciences (SIMATS) Saveetha University, Chennai, IND

**Keywords:** diabetic diet, non-communicable disease, physical activity, urban population, summary of diabetes self-care activities (sdsca)

## Abstract

Background

Diabetes is a long-term medical condition characterized by consistently high blood glucose levels, which can be attributed to both genetic and environmental factors. Global diabetes prevalence is predicted to reach 10.4% by 2040, impacting over 642 million people. Diabetes is becoming more common in India; by 2030, an estimated 87 million individuals are predicted to have the disease. Self-care activities are essential for managing diabetes, yet adherence to these practices is often low in developing countries like India due to socioeconomic, cultural, and healthcare barriers.

Objective

In this study, the self-care practices of diabetic patients who visited an Urban Health Center in Thirumazhisai, Tamil Nadu, were assessed, along with the factors influencing these practices.

Methods

A three-month cross-sectional study was conducted in a facility with 200 type 2 diabetic patients who had been unwell, for a minimum of a year. The participants were selected via purposeful sampling, and interviews were conducted using the most recent version of the Summary Diabetes Self-Care Activities (SDSCA) questionnaire. The data was analyzed using SPSS version 26 (IBM Corp., Armonk, NY, USA) to discover connections between the duration of diabetes and self-care practices using descriptive statistics and chi-square tests.

Results

The study found that 136 (68%) of participants tested their blood sugar levels at least once in the preceding three months, 104 (52%) exercised for at least 30 minutes each day, and 96 (48%) of individuals maintained a balanced diet. On the other hand, adherence to insulin injections and oral hypoglycemic medications was lower, at 98 (49%) and 79 (39.5%), respectively. Foot hygiene was also not at its best; only 85 (42.5%) people washed their feet daily. Considerable correlations have been shown between the length of diabetes and particular self-care behaviors, including eating patterns, exercise routines, and foot hygiene.

Conclusion

The results emphasize the necessity of focused interventions, especially in developing nations, to enhance diabetic patients' self-care behaviors. Tailored, patient-centered strategies are essential to address the complex factors influencing diabetes management, ultimately improving glycemic control and patient outcomes.

## Introduction

Diabetes is characterized by persistently elevated levels of glucose in the blood, which manifest themselves as a result of a confluence of environmental and genetic variables. A combination of genetic and environmental factors causes this condition. It is projected that 642 million people worldwide will be affected by diabetes by 2040 when the disease will be 10.4% more common. It was projected that 422 million adults worldwide would have diabetes in 2016, accounting for 8.8% of cases [1]. When compared to other countries, India has the greatest prevalence of diabetes.

As per the International Diabetes Federation (IDF), the number of individuals in India with a diabetes diagnosis was 69.2 million in 2015. It is projected that by 2030, this figure will increase to 87 million. The increase in the occurrence of the condition is associated with urbanization, industrialization, and the lifestyle change that has taken place in India [2]. Diabetes is associated with a higher risk of cardiovascular disease, stroke, and renal failure.

Activities that are considered to constitute diabetic self-care are those that are carried out by people who have diabetes or those who are at risk of developing the condition to control the disease on their own effectively. Individuals who have diabetes who engage in seven essential self-care activities are more likely to experience excellent outcomes. The following habits have been linked to improved quality of life, reduced complications, and glucose control: regular medication intake, physical exercise, healthy food, blood sugar monitoring, problem-solving and coping skills, and risk-reduction practice [3]. It has been reported that there is a low level of adherence to treatment regimens in the Indian social and cultural environment. This is attributed to low health knowledge and negative attitudes toward the illness. Self-care is difficult for people in developing countries like India due to the lack of access to pharmaceuticals, the high cost of medications, the unequal distribution of healthcare providers in rural and urban areas, and persisting cultural barriers.

Self-care practices taken by people who have diabetes or who are at risk of getting diabetes to effectively control the condition on their own are referred to as diabetes self-care activities. Individuals who have diabetes can predict positive outcomes by engaging in seven essential activities related to self-care. Good diet, exercise, frequent medication administration, blood sugar monitoring, coping mechanisms, problem-solving skills, and risk-taking are all associated with improved quality of life, reduced complications, and glucose control [4-8]. As a result of inadequate health literacy and unfavorable attitudes toward the condition, it has been shown that there is a low level of compliance with treatment regimens in the Indian sociocultural environment. In persons belonging to lower socioeconomic strata, non-compliance was shown to be more widespread [9]. The inequitable distribution of health professionals between urban and rural areas, the high cost of medications, their restricted availability, and prevailing cultural obstacles all hinder the practice of self-care in developing countries like India [4,10]. In this study, adult patients with type 2 diabetes who were located in metropolitan southern India were asked to evaluate their current self-care practices as well as the factors influencing these practices.

## Materials and methods

This study was conducted at the Urban Health Center in Thirumazhisai, a panchayat town in Poonamalle Taluk, inside the Thiruvallur district of Tamil Nadu, India. It was designed as a facility-based cross-sectional study. A total of 19,733 people live in Thirumazhisai, which has a land area of around six square kilometers, according to the 2001 census. Three months were spent doing the study.

Patients with Type 2 Diabetes Mellitus (T2DM) for at least a year were the study's target population. Participants had to meet the inclusion criteria, which included having T2DM for at least a year and being prepared to give informed permission. Individuals who were hesitant to participate in the trial or had systemic or localized chronic diabetes problems were excluded. Purposive sampling, a non-probability sampling strategy, was used to choose participants to concentrate on people who fit particular requirements pertinent to the study's goals.

Utilizing the formula (4PQ/L2), the sample size for the study was established while accounting for a previous investigation that found 85% of patients with Type 2 Diabetes Mellitus (T2DM) engaged in self-care activities. The symbols in this formula are (Q) for (1-P), (L) for allowable error, and (P) for prevalence. According to calculations, 200 participants with a 95% confidence interval and a 5% alpha error would be needed for this study [11].

**Figure 1:**
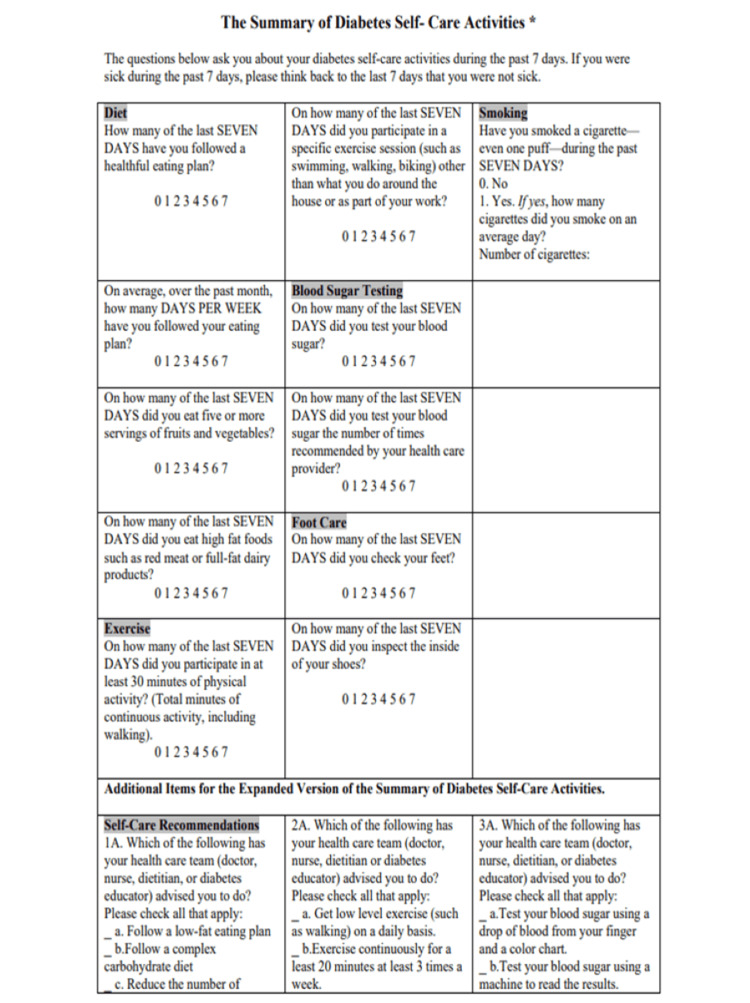
Self-care component of diabetes patients

**Figure 2:**
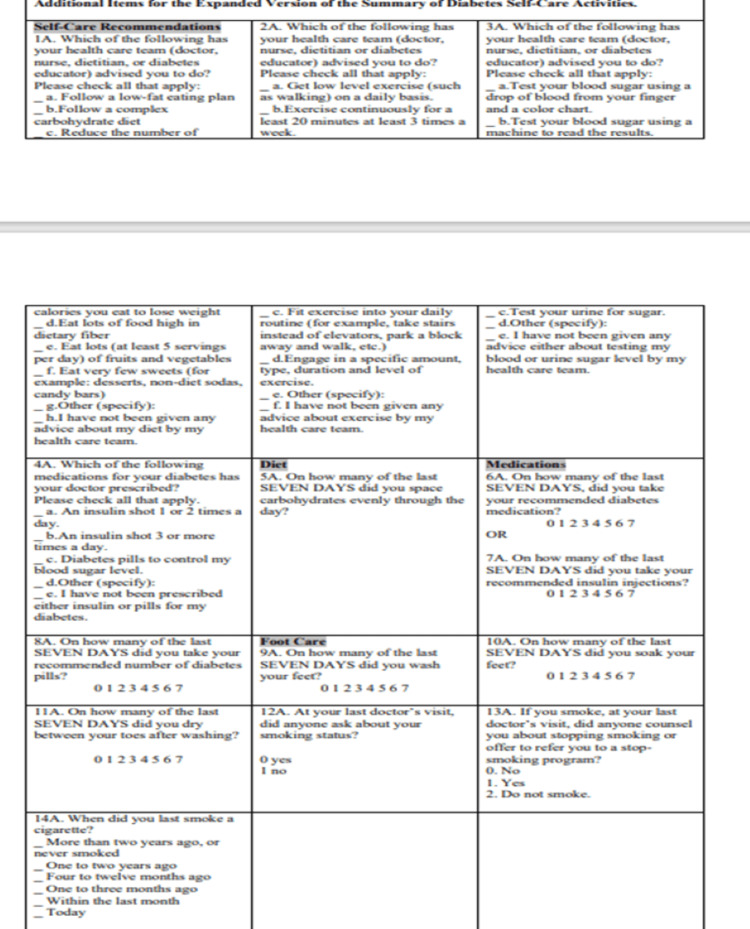
Additional items for the expanded version of the summary of diabetes self-care activities

**Figure 3:**
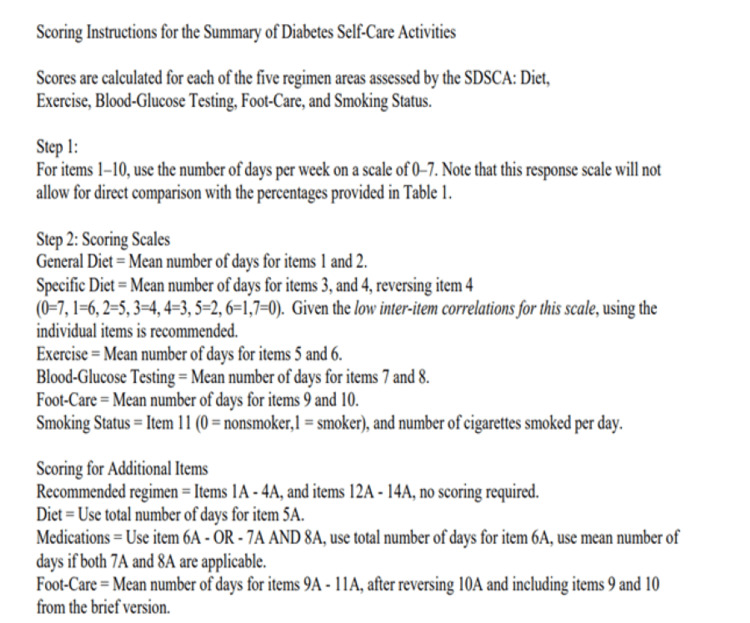
Scoring instructions for the summary of diabetes self-care activities

Ethical approval had been granted by the Saveetha Medical College and Hospital (SMCH) Institutional Ethics Committee (101/07/2023/IEC/SMCH). The procedure of gathering data involved the use of two primary tools. Sociodemographic data, including age, gender, marital status, literacy level, alcohol intake, socioeconomic status, and random blood sugar levels, were first gathered via a pre-test semi-structured questionnaire. The Revised Summary of Diabetes Self-Care Activities (SDSCA) questionnaire served as the second tool [12]. The questionnaire has been attached in the Appendices (Figures 1-3).

There was another component that determined medication adherence. Each component was scored on an ordinal scale from 0 to 7, with higher scores indicating greater self-care practices. The SDSCA, a validated measure in diabetes research, assesses five primary categories of self-care behaviors: diet, exercise, blood sugar testing, foot care, and smoking.

Data were gathered through in-person interviews conducted by licensed healthcare professionals. Except for blood sugar testing, which was assessed throughout the preceding three months, all self-care practices were asked to be recalled from the previous week. To ensure that all relevant information was collected efficiently and methodically, concurrent socio-demographic data collection was carried out during these interviews.

Microsoft Excel was used to collect and store the gathered data in an initial manner. Version 26 of SPSS (IBM Corp., Armonk, NY, USA) was used for the analysis that followed. To summarize participant characteristics and self-care habits, descriptive statistics were computed, such as means, standard deviations, frequencies, and percentages. The Chi-square test was used to find significant connections between categorical characteristics and reported self-care behaviors to investigate the relationships between sociodemographic variables, the length of diabetes, and self-care activities.

## Results

The presented data outlines key variables related to individuals with diabetes mellitus (DM) and examines their glycemic control and various demographic and health-related factors. The study includes age, gender, socio-economic status (SE status), family history of DM, education, occupation, alcohol consumption, age of onset of DM, duration of DM, blood glucose monitoring, and glycemic control. The age distribution reveals that 20 individuals are below 40, while 180 are above 40. Gender-wise, there are 90 males and 110 females in the study population. The socio-economic status (SE status) is categorized into five classes (I-V), with the majority falling into classes II and III. Regarding family history of DM, seven individuals have both parents affected, 71 have a single parent affected, and 122 report no family history of DM. Education status highlights that 138 individuals are illiterate, while 62 are literate. Occupationally, the study participants are engaged in various roles, including dependent work, daily wages, employment, business, merchant, retirement, and homemaking. In terms of alcohol consumption, 20 are current drinkers, 60 are past drinkers, and 120 have never consumed alcohol. The age of onset of DM is divided into two categories, with 44 individuals experiencing onset before 40 years and 164 having onset at or after 40 years. Table 1 shows the duration of DM which reveals that 151 individuals have had DM for 10 years or less, while 49 have had it for more than 10 years. The majority, 136 (68%) individuals, report blood glucose monitoring practices.

**Table 1:** Sociodemographic details of the study participants *Modified BG Prasad classification H/O- History of; DM- Diabetes mellitus

VARIABLES		FREQUENCY (N=200)	PERCENTAGE (%)
Age	<40 years	20	10
	>40 years	180	90
Sex	Male	90	45
	Female	110	55
Socio-economic status	Class I	30	15
	Class II	62	31
	Class III	60	30
	Class IV	28	14
	Class V	20	10
Family H/O DM	Both parents	7	3
	Single parent	71	36
	No	122	61
Education	Illiterate	138	69
	Literate	62	31
Occupation	Dependant	10	5
	Daily wages	38	19
	Employee	38	19
	Business	4	2
	Merchant	25	12
	Retired	23	11
	Homemaker	52	26
Alcohol consumption	Current drinker	20	10
	Past drinker	60	30
	Never	120	60
Age of onset of DM	<40 years	44	22
	≥40 years	164	82
Duration of DM	≤10 years	151	75
	>10 years	49	24
Blood glucose monitoring (once in 3 months)	Yes	136	68
	No	64	32
Glycemic control	Yes	109	54
	No	91	46

Table 2 represents the various self-care behaviors and their adherence frequencies within a specific population. Among the self-care practices, most individuals (114, 57%) walk barefoot daily. This high frequency could indicate a lack of awareness about the risks of walking barefoot, especially for individuals with diabetes or other conditions that increase susceptibility to foot injuries and infections. Walking barefoot can expose individuals to various hazards such as cuts, infections, and other foot injuries, which can be particularly dangerous for those with compromised foot health or reduced foot sensation. This practice highlights the need for education and intervention to promote better foot care habits, particularly among vulnerable populations.

**Table 2:** Self-care practices among diabetic patients

Self-care component	Frequency (%)
Following a healthy eating plan on all days of the week	96(48%)
Incorporating fruits/vegetables in the diets on all days of the week	111(55.5%)
Consumption of fried food products on all days of the week	78(39%)
Consumption of high-fat diet on all days of the week	27(13.5%)
At least 30 minutes of physical activity daily	104(52%)
Specific exercise sessions apart from the routine physical activity daily	30(15%)
Blood sugar testing at least once in the past 3 months	136(68%)
Adherence to oral hypoglycemic agents on all days of the week	79(39.5%)
Adherence to insulin injections on all days of the week	98(49%)
Washing feet on all days of the week	85(42.5%)
Drying in between the toes on all days of the week	58(29.0%)
Examining feet on all days of the week	78(39.0%)
Walking in barefoot	114(57%)

The following tables (Tables 3-6) represent the association between self-care practices and outcomes in individuals with diabetes based on the duration of their condition: less than 10 years and more than 10 years. Following a healthy eating plan and adherence to a healthy eating plan do not significantly differ between those with diabetes for less than 10 years and those with diabetes for more than 10 years (p = 0.386). Incorporating fruits/vegetables in the diet in this study shows that there is no significant difference in incorporating fruits and vegetables in the diet between the two groups (p = 0.437). Consumption of fried food products shows that it differs significantly between the groups, with a higher percentage in the <10 years group (p = 0.01). Consumption of high-fat diet shows that there is a significant difference in the consumption of a high-fat diet, with more individuals in the <10 years group consuming high-fat diets (p = 0.043). There is a significant difference observed in engaging in at least 30 minutes of physical activity daily, with the groups having equal participation but overall significant impact (p = 0.001). A significant difference was observed in engaging in specific exercise sessions apart from routine physical activity, with more individuals in the <10 years group participating (p = 0.01). Significant differences were also observed in adherence to oral hypoglycemic agents, foot care practices, and walking barefoot. These findings highlight the need for targeted interventions based on the duration of diabetes to improve health behaviors and outcomes.

**Table 3:** Association between diet component of self-care practices and duration since diagnosis of diabetes *p-value < 0.05 is significant.

VARIABLES	SUBCATEGORIES	DURATION OF DIABETES <10 Years	DURATION OF DIABETES >10 Years	P-VALUE
Following a healthy eating plan on all days of the week?	Yes	46(47.9%)	50(52%)	0.386
	No	53(50%)	51(49%)	
Incorporating fruits/vegetables in the diets on all days of the week	Yes	56(50.4%)	55(49.5%)	0.437
	No	43(48.3%)	4651.6%)	
Consumption of fried food products on all days of the week	Yes	47(60.2%)	31(39.7%)	0.01*
	No	52(42.6%)	70(57.3%)	
Consumption of high-fat diet on all days of the week	Yes	18(66.6%)	9(33.3%)	0.043*
	No	81(46.8%)	92(53.1%)	

**Table 4:** Association between physical activity practices and duration since diagnosis of diabetes *p-value < 0.05 is significant.

VARIABLES	SUBCATEGORIES	DURATION OF DIABETES <10 Years	DURATION OF DIABETES >10 Years	P-VALUE
At least 30 minutes of physical activity on a daily basis	Yes	52(50%)	52(50%)	0.001*
	No	47(48.9%)	49(51%)	
Specific exercise session apart from the routine physical activity on a daily basis	Yes	21(70%)	9(30%)	0.01*
	No	78(45.8%)	92(54.1%)	

**Table 5:** Association between blood sugar monitoring and duration since diagnosis of diabetes *p-value < 0.05 is significant.

VARIABLES	SUBCATEGORIES	DURATION OF DIABETES <10 Years	DURATION OF DIABETES >10 Years	P-VALUE
Blood sugar testing at least for once in the past 3 months	Yes	64(47%)	72(52.9%)	0.19
	No	35(54.6%)	29(45.3%)	
Adherence to oral hypoglycemic agents on all days of the week	Yes	30(37.9%)	49(62%)	0.006*
	No	69(57%)	52(42.9%)	

**Table 6:** Association between foot care and duration since diagnosis of diabetes *p-value < 0.05  is significant.

VARIABLES	SUBCATEGORIES	DURATION OF DIABETES <10 Years	DURATION OF DIABETES >10 Years	P-VALUE
Washing feet on all days of the week	Yes	61(71.7%)	24(28.2%)	0.01*
	No	38(33%)	77(66.9%)	
Drying in between the toes on all days of the week	Yes	39(67.2%)	19(32.7%)	0.01*
	No	60(42.2%)	82(57.7%)	
Examining feet on all days of the week	Yes	51(65.3%)	27(34.6%)	0.002*
	No	48(39.3%)	74(60.6%)	
Walking in barefoot	Yes	69(60.5%)	45(39.4%)	0.002*
	No	30(34.8%)	56(65.1%)	

In summary, the non-significant associations observed in this study suggest that factors such as the duration of DM, education level, family history of DM, and blood glucose monitoring practices may not be singularly influential in predicting glycemic control outcomes among individuals with diabetes. To achieve optimal glycemic control, a comprehensive and personalized approach that takes into account a variety of parameters is necessary, as these findings highlight the complicated aspects of diabetes care.

## Discussion

The study assessed how patients using a primary care hospital were implementing diabetes self-care activities. Of the study participants, 48% follow a healthy eating plan every day of the week on average. Similar results have been reported by Padma et al. [13]. Adhering to a consistent dietary plan, focusing on both portion size and nutritional value, is crucial for maintaining optimal blood sugar levels and effective weight control. Approximately 55.5% of research participants consumed solely vegetables and fruits every day of the week. A misperception that eating fruit can raise blood sugar levels and a lack of knowledge about the importance of eating fruits and vegetables may be the cause of the low intake in our study. It is recommended by the World Health Organization (WHO) to consume 400 grams or more of fruits and vegetables every day. Consuming enough fruits and vegetables helps control blood sugar levels and reduces the risk of heart disease, stroke, and gastrointestinal malignancies [14]. It was reassuring to observe that only a small percentage of participants (15.8%) consistently had a high-fat diet every day of the week. Gopichandran et al.’s results show that [15].

Only 52% of study participants exercised for 30 minutes a day, and 15% added an extra exercise session to their regular regimen, indicating an inadequate commitment to the physical activity component of self-care activities. Rough findings from studies conducted in India and other nations were reported [16-18]. Most (68%) of the study participants monitored their blood sugar levels at least once every three months. Comparable results were noted in investigations carried out in other locations. Consistent monitoring of blood glucose levels is crucial for managing diabetes as it aids in evaluating the efficacy of the patient's current treatment plan. Since home blood glucose monitoring is not common in the study area, it was not assessed in our study. Our study participants showed adherence rates of 39.5% to oral hypoglycemic medications and 49% to insulin injections. Gopichandran et al.’s [15] study showed an adherence rate of 79%. The conclusion could be attributed to healthcare providers' frequent health education messages, emphasizing that strict adherence to prescriptions can prevent or reduce acute or chronic diabetes problems.

Most of the individuals in our study engaged in the daily practice of washing their feet (42.5%) and evaluating their feet (39%). In a study conducted in Pakistan, only 20% of individuals washed their feet daily and 17% inspected their feet daily, which contrasts with the current conclusion [19]. The variation could result from the custom of regular foot washing in India for religious and cultural purposes. Nevertheless, our study revealed a low adherence to crucial foot care practices, such as drying between toes and inspecting the inner surface of shoes for any discharge, which aligns with similar observations in studies from Pakistan and Sri Lanka [20]. Foot care practices are crucial for preventing foot ulcers and the progression to gangrenous lesions, which may necessitate limb amputations and lead to greater disability and impairment. In Tanzania, a study revealed that those who got foot care guidance from doctors were more inclined to engage in foot care compared to those who did not receive such advice. The association between glycemic control and factors such as diabetes duration, education level, blood glucose monitoring, and family history of diabetes was insignificant, contradicting the results of a study conducted in Bangalore [21,22]. Diabetic patients should focus on self-care behaviors and glycemic management to improve outcomes and quality of life.

Limitation

One limitation of this study is the reliance on RBS values to determine glycemic control. The HBA1c number, used to assess blood sugar control, was not utilized due to financial constraints among the majority of the participants. Secondly, our study was cross-sectional and conducted exclusively inside a hospital setting. Participants were individuals who visited the outpatient clinic at the primary health center. There may be a significant number of diabetic individuals in the community who have not been to a hospital for a follow-up appointment, and their blood sugar levels may vary. Thirdly, our sampling method was based on convenience.

## Conclusions

In conclusion, the comprehensive analysis of variables related to glycemic control in individuals with diabetes presents a nuanced understanding of the multifactorial nature of diabetes management. The results of this study indicate that, in comparison to those who have had diabetes for less than 10 years, those who have had the disease for more than 10 years often have better eating habits and medication adherence, but they may also participate in less regular physical activity and foot care. People were more likely to adopt healthier eating habits as a result of the strong connections, maybe as a result of increasing awareness of the role that diet plays in managing diabetes. These findings are significant because people with diabetes who have had the disease for a longer period have had more time to establish and stick to medication regimens, which improves adherence. Additionally, because people with longer-duration diabetes are more likely to experience complications, foot care must be taken more carefully. Results that are not statistically significant point to regions where behavior remains constant for people with diabetes, irrespective of their duration of illness. The findings contribute valuable insights, emphasizing health care providers can help individuals with diabetes, regardless of the duration of their condition, improve their overall management and reduce the risk of complications. Early and continuous support is crucial to fostering long-term healthy habits that can lead to better outcomes for people living with diabetes.
